# Genome-Wide Analysis of *Mycoplasma bovirhinis* GS01 Reveals Potential Virulence Factors and Phylogenetic Relationships

**DOI:** 10.1534/g3.118.200018

**Published:** 2018-03-30

**Authors:** Shengli Chen, Huafang Hao, Ping Zhao, Yongsheng Liu, Yuefeng Chu

**Affiliations:** State Key Laboratory of Veterinary Etiological Biology, Lanzhou Veterinary Research Institute, Chinese Academy of Agricultural Sciences, Xujiaping 1, Lanzhou, 730046, Gansu, People’s Republic of China

**Keywords:** *Mycoplasma bovirhinis*, genome, virulence gene, comparative analysis, phylogenetic analysis, Genome Report

## Abstract

*Mycoplasma bovirhinis* is a significant etiology in bovine pneumonia and mastitis, but our knowledge about the genetic and pathogenic mechanisms of *M. bovirhinis* is very limited. In this study, we sequenced the complete genome of *M. bovirhinis* strain GS01 isolated from the nasal swab of pneumonic calves in Gansu, China, and we found that its genome forms a 847,985 bp single circular chromosome with a GC content of 27.57% and with 707 protein-coding genes. The putative virulence determinants of *M. bovirhinis* were then analyzed. Results showed that three genomic islands and 16 putative virulence genes, including one adhesion gene enolase, seven surface lipoproteins, proteins involved in glycerol metabolism, and cation transporters, might be potential virulence factors. Glycerol and pyruvate metabolic pathways were defective. Comparative analysis revealed remarkable genome variations between GS01 and a recently reported HAZ141_2 strain, and extremely low homology with others mycoplasma species. Phylogenetic analysis demonstrated that *M. bovirhinis* was most genetically close to *M. canis*, distant from other bovine *Mycoplasma* species. Genomic dissection may provide useful information on the pathogenic mechanisms and genetics of *M. bovirhinis*.

Bovine respiratory disease (BRD) and cow mastitis have caused considerable economic losses worldwide, and many microbial pathogens, including viruses and bacteria, have contributed to such losses (Griffin *et al.* 2010; Mosier 2014; Fox 2012). In addition, *Mycoplasma* species play a major role in the onset of these diseases (Srikumaran *et al.* 2007). More than 12 *Mycoplasma* species, such as *M. bovis*, *M. bovigenitalium*, *M. dispar*, and *M. bovirhinis*, have been reported to be involved in BRD and mastitis (Fox 2012; Martin 1978). *M. bovirhinis* is one of the most commonly occurring species in respiratory diseases and bovine mastitis in many countries (ter Laak *et al.* 1992; Ayling *et al.* 2004). In respiratory diseases, *M. bovirhinis* is normally regarded as a secondary invader that can co-infect with other pathogens and aggravate calf pneumonia (Miles *et al.* 2004).

*M. bovirhinis*, belonging to *Mycoplasmataceae* under *Mollicutes*, has a relatively small genome and has no cell wall. It was first isolated by Harborne in mastitis cows in England in 1965 (Harbourne *et al.* 1965). *M. bovirhinis* concurrently infects calves with other etiologies and causes respiratory diseases (Hirose *et al.* 2003), mastitis (Higuchi *et al.* 2011), and otitis (Lamm *et al.* 2004). This pathogen is usually isolated from the nasal mucus (Hirose *et al.* 2003) and lung (Soehnlen *et al.* 2011) of pneumonic calves or from calves with mastitis (Fox 2012), otitis (Lamm *et al.* 2004), and subacute nephritis (Panangala *et al.* 1990). *M. bovirhinis* is occasionally detected in clinically healthy calves, but its detection rate in such calves is lower than that in pneumonic calves (Angen *et al.* 2009). Despite epidemiological and antimicrobial susceptibility investigations on *M. bovirhinis*, the pathogenesis of this pathogen remains unknown.

In 2016, a severely contagious respiratory disease with an incidence of more than 50% and mostly affected calves spread in a dairy farm in Gansu Province in China. The main symptoms recorded were cough, asthma, high fever, and emaciation. Nasal swabs were sampled and examined through conventional isolation and molecular identification. One *Mycoplasma* strain named GS01 was isolated from the samples with an improved Friis medium, but other pathogenic bacteria were not detected. After cloning purification was performed thrice, 16S rRNA sequence and specific PCR analysis (Kobayashi *et al.* 1998) further confirmed that the isolate is *M. bovirhinis*.

Many *Mycoplasma* species have been identified and sequenced genomically. At present, the virulence factors and evolutionary relationships of *M. bovirhinis* are still poorly understood although the genome of a Japanese strain HAZ141_2 was recently reported (Hata *et al.* 2017). In the present study, we report the complete genomic sequence of *M. bovirhinis* strain GS01, and identify putative relevant virulence factors. Comparative and phylogenetic analyses are also conducted. The data presented in this study may improve our current understanding on the pathogenic mechanisms and genetics of *M. bovirhinis*.

## MATERIALS AND METHODS

### Bacterial Culture and DNA Preparation

The GS01 strain was isolated with a deep nasal swab in a modified Friis medium (Friis powder 21.4 g/L, glucose 2 g/L, 10% horse serum, 10% porcine serum, 100 mg/L ampicillin sodium, 0.01% acetic acid thallium, adding 1 M sodium hydroxide adjust to pH 7.4) at 37° for 2 days. The colony was cultured on an agar plate (adding 1.5% agar in the modified Friis medium) at 37° in a 5% CO_2_ atmosphere for 5 days. The strain was purified thrice. A 500 mL mid-exponential phase culture was pelleted through centrifugation at 10,000 × g for 20 min and subjected to centrifugal washing with PBS (0.01 M, pH 7.2) twice. Total genomic DNA was extracted and treated with RNase by using a TIANamp bacterial DNA kit (Tiangen, Beijing, China) according to the manufacturer’s instructions.

### Library Construction and DNA Sequencing

The genomic DNA was detected through agarose gel electrophoresis and was used to prepare a 10-kb size-selected PacBio SMRTbell libraries following the manufacturer’s instructions. After purification was performed, the libraries were quantified by Qubit, and the insertion size was detected. The genome of *M. bovirhinis* GS01 was sequenced using the PacBio RSII platform, resulting in a 1026-fold sequencing depth and 719× depth of coverage. Genome sequencing was conducted at the Beijing Novogene Bioinformatics Technology Co., Ltd.

### Genome Assembly and Annotation

Low-quality raw data were filtered by SMRT v2.3.0 analysis software suite to obtain clean data. A total of 83,447 reads totaling 1,023,037,568 bases (mean read length: 12,259 bp) was obtained. The N50 read length was 15,812 bp and mean read score was 0.82. Read length distribution for the sequenced genome is shown in Figure S1 in File S1. Genome assembly was performed using the SMRT portal, included in SMRT v2.3.0 and the *de novo* assembly was conducted following the hierarchical genome-assembly process (HGAP) assembly protocol with Quiver polishing (Chin *et al.* 2013). Finally, one polished contig without gap was generated. The depth of coverage distribution map for genome assembly is shown in Figure S2 in File S1.

The genome component prediction was conducted as follows: gene prediction was conducted using GeneMarkS v4.17 (Besemer *et al.* 2001) with default parameters. Transfer RNAs (tRNAs) were identified using tRNAscan-SE v1.3.1 (Lowe and Eddy 1997) with default parameters. Ribosomal RNAs (rRNAs) were detected using RNAmmer v1.2 (Lagesen *et al.* 2007) with default parameters. Small nuclear RNAs (snRNAs) were identified using BLAST against the Rfam v12.1 (Gardner *et al.* 2009). Pseudogenes were predicted by the NCBI Prokaryotic Genome Annotation Pipeline on the GenBank database. Genomic islands, insertion sequences, interspersed repetitive sequences, and tandem repeats were predicted with IslandPath-DIOMB program (Hsiao *et al.* 2003), ISfinder (https://www-is.biotoul.fr/), RepeatMasker v4.0.5 (Saha *et al.* 2008), and TRF v4.0.7b (Benson 1999) with default parameters, respectively.

Functional annotations were conducted by BLASTP algorithm similarity search against the non-redundant (NR) protein database (release 2016-04), Swiss-Prot (release 2016-04), Clusters of Orthologous Groups (COG) (release 2015-12-14), Gene Ontology (GO) (release 2014-10-19), and Kyoto Encyclopedia of Genes and Genomes (KEGG) databases (release 2016-04). BLASTP algorithm were set as E-value less than 1e-5, and the homology identity and minimal alignment length percentage were larger than 40% for the above functional annotation tools. The results were subsequently filtered by selecting the highest score of the alignment. The potential virulence genes were predicted through gene annotation and reference studies (Bürki *et al.* 2015; Chen *et al.* 2011; Hames *et al.* 2009; Groisman *et al.* 2013; Zheng *et al.* 2015). Secretory proteins were predicted with the SignalP v4.1 database (Petersen *et al.* 2011) with default parameters, and type III effector proteins secreted by *M. bovirhinis* GS01 were predicted with EffectiveT3 (Eichinger *et al.* 2016) with default parameters.

### Comparative and Phylogenetic Analysis

The genome comparisons between *M. bovirhinis* GS01 and HAZ141_2 were conducted using the above genome annotation methods. Genomic synteny was analyzed on the basis of the results of the alignment, which was conducted using MUMmer v3.23 (Delcher *et al.* 2003) and LASTZ v1.03.54 (Chiaromonte *et al.* 2002) tools between GS01 and referenced HAZ141_2 genome under default parameters. Briefly, the large scale co-linear relationship of the target GS01 genome and the referenced HAZ141_2 genome was determined using mummer program under MUMmer v3.23 software package with default parameters. The output of results were then processed regionally by LASTZ v1.03.54 with best-chain alignments following the manufacturer’s instructions, and the local arrangement of relationships (collinear, translocation, inversion and translocation + inversion) were determined. The presented results are derived from a combination of MUMmer and LASTZ analyses. The overall sequence similarity of the two genomes was calculated using BLASTN v2.2.26 alignment method. Core genes were identified by CD-HIT software v4.6 (Li and Godzik 2006), with the following parameters: 50% pairwise identity threshold and 0.7 length difference cutoff in amino acid. Multiple sequences of single-copy core genes among 19 *Mycoplasma* strains were aligned using MUSCLE v3.8.31 (Edgar 2004). Phylogenetic trees based on the single-copy core genes were constructed by TreeBeST v1.9.2 (Nandi *et al.* 2010) using the maximum likelihood model with 1000 bootstrap replicates under default parameters and by MrBayes v.3.2.6 (Ronquist *et al.* 2012) using Bayesian inference method. Bayesian inference phylogenyetic construction was conducted using mixed model under the following set conditions: mcmc ngen = 2 × 10^4^, samplefreq = 100, printfreq = 1000, diagnfreq = 1000, and to obtain standard deviation of split frequencies below 0.01. After discarding the burn-in samples, a Bayesian phylogenyetic tree was generated based on the remaining data and was shown by using iTOL tool (http://itol.embl.de/). The genome sequence of other *Mycoplasma* species were obtained from the NCBI database.

### Data availability

Strains are available upon request. The genome sequence data were deposited in GenBank with the accession number CP024049. Supplemental Material, Figure S1 in File S1 shows PacBio read length distribution for the sequenced *M. bovirhinis* GS01 genome. Figure S2 in File S1 shows the depth of coverage distribution map for *M. bovirhinis* GS01 genome assembly. Table S1 in File S1 shows prediction of the genome component of *M. bovirhinis* GS01. Table S2 in File S1 shows the statistical results of repetitive sequences of *M. bovirhinis* GS01. Table S3 in File S1 shows functional category in COG of *M. bovirhinis*. Table S4 in File S1 shows genes predicted involving in transporter system of *M. bovirhinis* GS01. Table S5 in File S1 shows the predicted genes involving in metabolism in the GS01 genome. Table S6 in File S1 shows proteins involved in secretion system of *M. bovirhinis* GS01. Table S7 in File S1 shows the effective proteins of T3SS in *M. bovirhinis* GS01 genome. Table S8 in File S1 shows genes in the genomic islands of *M. bovirhinis* GS01. Table S9 in File S1 shows the genome comparisons between *M. bovirhinis* GS01 and HAZ141_2 strains. Table S10 in File S1 shows the genes of the 53.4-kb deletion in GS01 relative to HAZ141_2. Table S11 in File S1 shows list of 14 single-copy core genes of 19 selected *Mycoplasma* strains.

## RESULTS

### General Genome Features

**Figure 1:**
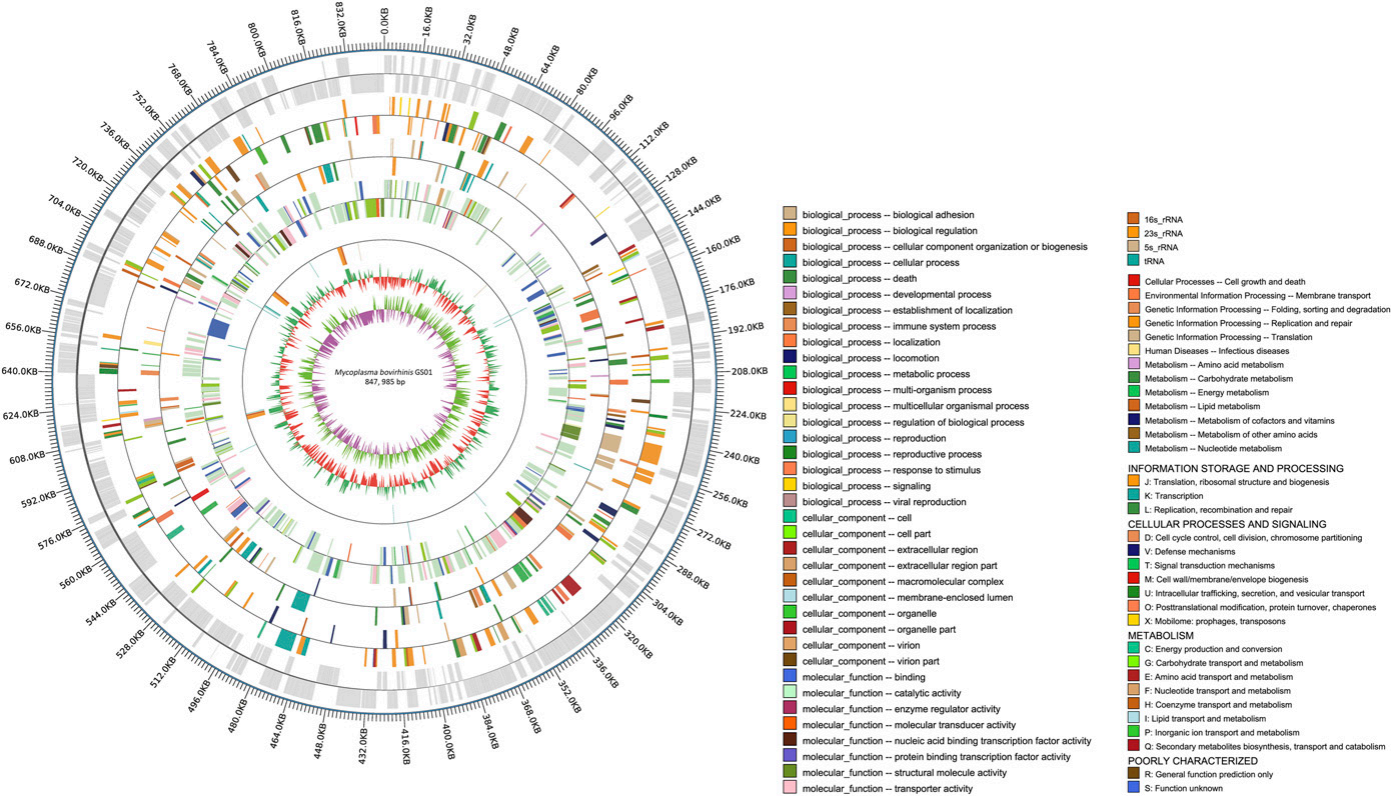
Chromosome atlas of *Mycoplasma bovirhinis* strain GS01. *dnaA* at position 1. From outer to inner circles, the first circle represents the genome position coordinates; the second circle shows the locations of predicted coding genes on plus and minus strands; the third, fourth, and fifth circles show the results of color-coded by COG categories, KEGG, and GO annotation (see the description at the bottom-right corner), respectively; the sixth circle represents the ncRNA in GS01 genome; the seventh circle represents the mean centered G+C content whose baseline is average GC, and outward and inward projections express higher and lower values than the average, respectively; and the eighth circle illustrates the GC (G+C) skew plot: above and below zero are highlighted in green and purple, respectively.

The complete genome of *M. bovirhinis* GS01 is composed of a 847,985 bp single circular chromosome with a 27.57% GC content (Figure 1). A total of 83,447 reads with an average length of 12,259 bp were produced. Then, 707 protein-encoding genes with an average length 1,072 bp were identified in the genome, and the coding percentage of the genome was 89.34%. The non-coding RNA of this organism consists of 31 tRNAs and 8 rRNAs (2 5S rRNA, 3 16S rRNA, and 3 23S rRNA) (Table S1 in File S1). Moreover, 67 tandem repeats and 68 interspersed nuclear elements were identified. Table S2 in File S1 presents the statistical results of the repetitive sequences of *M. bovirhinis* GS01.

Of the 707 coding genes of *M. bovirhinis* GS01, 259 were assigned to 20 functional categories in COG database (Table S3 in File S1) and 81 (31.27%) were involved in transport and metabolism. Biological functions were defined for 462 (65.34%) genes, and 127 genes encoded hypothetical proteins with unknown functions. Genomic sequence data were submitted to GenBank with the accession number CP024049.

### Transporter and Metabolism

A total of 56 *M. bovirhinis* genes were annotated as being related to transporter systems (Table S4 in File S1). These mainly belonged to the ATP-binding cassette (ABC) transporter system and the phosphotransferase system (PTS). Furthermore, 42 genes were encoded for the ABC transporter system, including 17 ATP-binding proteins, 16 permease proteins, two substrate-binding proteins, and seven other proteins. These transporters are mainly involved in maltose/maltodextrin, cobalt/nickel, spermidine/putrescine, oligopeptide, sugar, phosphate/phosphonate, and cation transport systems. The PTS system contained six genes, including *ptsI* and *ptsH*, and four genes encoded carbohydrate-specific EII complexes, which catalyze concomitant carbohydrate translocation and phosphorylation. The usually three-component EII complex was composed of hydrophilic IIA and IIB and carbohydrate-selective transmembrane IIC domains. The GS01 genome has one fructose-specific, one lichenan-specific, and two glucose-specific EIIABC components, which indicated that the PTS system is involved in the saccharide absorbance of *M. bovirhinis*.

Given their small genomes, *Mycoplasma* have lost most biosynthetic activities and lack many metabolic pathways, forcing them to obtain nourishment from their host. Although 91 genes were predicted to participate in the metabolic system of *M. bovirhinis* (Table S5 in File S1), the metabolic ability was defective. The glycerol glycerophosphocholine importer GlpU, oxidase GlpO, and glycerol import system GtsABC were not found in the genome. Glycerol uptake may be facilitated by the glycerol uptake facilitator protein GlpF (Mbr-GS01GM000491) and phosphorylated by glycerol kinase GlpK (Mbr-GS01GM000492) to become glycerol-3-phosphate (G3P), which is transformed into glyceraldehyde-3-phosphate by glycerol-3-phosphate dehydrogenase GlpD (Mbr-GS01GM000493) and triosephosphate isomerase (Mbr-GS01GM000512). Glyceraldehyde-3-phosphate is an important product of the glycolysis pathway and is metabolized into pyruvate. The genes required to transform glucose into pyruvate and pyruvate into lactate were identified in *M. bovirhinis*. However, the genes involved in tricarboxylic acid cycle were missing, such as pyruvate dehydrogenase (PDH) enzyme complex genes and phosphate acetyltransferase. Besides, two genes (Mbr-GS01GM000577 and Mbr-GS01GM000638) in the pentose phosphate pathway were also annotated in the genome.

### Secretion Systems

A total of 47 secreted proteins with N-terminal signal peptides were predicted in the GS01 genome, and the peptides were 19–31 amino acids long. Our results revealed the components of the secretion machinery, which included the signal recognition particle receptor FtsY and subunit Ffh; components SecA, SecE, SecG, SecY, and SecDF in the major translocation pathway and the general chaperone trigger factor DnaK, LepA; and the competence protein ComEA (Table S6 in File S1). There was one putative inner membrane protein translocase component YidC that is involved in the insertion of hydrophobic sequences into the lipid bilayer either independently or via the SecYEG translocase complex (Scotti *et al.* 2000). Moreover, one signal peptidase I gene and one signal peptidase II gene, which respectively encode the enzymes for the cleavage of the common protein and lipoprotein signal peptides, were found in the GS01 genome.

Furthermore, the effector proteins of type III secretion/translocation systems were predicted and 16 proteins (Table S7 in File S1), including ATP synthase, ribosomal protein, transporter protein, and a conversed hypothetical protein, were found. These proteins were secreted into the extracellular environment or host, which may be related to the survival and pathogenicity of bacteria.

### Virulence Factors

Three genomic islands were found in the GS01 genome (from 463,870 to 481,877, from 561,137 to 565,821, and from 597,644 to 607,572) with a total length of 32,622 bp, containing a total of 23 genes (Table S8 in File S1). No complete insertion sequence element was found in the genome, and seven transposes were found to locate outside the genomic islands. Although the particular function of proteins cited in the genomic islands were unclear, pathogenicity islands were often considered essential for bacteria virulence (Schmidt and Hensel 2004).

Adherence to host cells is a key step in *Mycoplasma* colonization and infection, and adherence proteins are regarded as virulence factors. Enolase is considered an adherence factor that contributes to adherence by binding a chicken plasminogen in *M. gallinaceum* (Chen *et al.* 2011). An enolase gene (Mbr-GS01GM000688) was identified in the GS01 genome (Table 1), which showed 87% amino acid identity with the α-enolase gene of *M. gallinaceum*, and might be associated with *M. bovirhinis* virulence. The capsule is also considered an important virulence factor in microorganisms, such as bacteria (Boyce and Adler 2000). Only one capsule synthesis-related gene (Mbr-GS01GM000446) was annotated in the genome and might be involved in the virulence of *M. bovirhinis*.

**Table 1:** Predicted virulence genes in the GS01 genome

Locus	Product	Gene	Protein length (aa)	Position
Mbr-GS01GM000045	membrane-associated lipoprotein	—	834	38324…40828
Mbr-GS01GM000101	predicted lipoprotein	—	345	113136…114173
Mbr-GS01GM000385	potassium transporter TrkA	*trkA*	223	431400…432071
Mbr-GS01GM000400	membrane-associated lipoprotein	—	833	456200…458701
Mbr-GS01GM000446	glycosyl transferase	—	340	522199…523221
Mbr-GS01GM000464	surface protein	—	713	545544…547685
Mbr-GS01GM000465	membrane-associated lipoprotein	—	851	547762…550317
Mbr-GS01GM000491	glycerol uptake facilitator protein	*glpF*	248	581842…582588
Mbr-GS01GM000492	glycerol kinase	*glpK*	505	582597…584114
Mbr-GS01GM000493	glycerol-3-phosphate dehydrogenase	—	384	584124…585278
Mbr-GS01GM000528	magnesium transporter	*mgtE*	524	621734…623308
Mbr-GS01GM000652	5′-nucleotidase	—	707	765862…767985
Mbr-GS01GM000661	magnesium-transporting ATPase (P-type)	*mgtA*	904	779505…782219
Mbr-GS01GM000688	enolase	*eno*	453	816615…817976
Mbr-GS01GM000690	P60-like lipoprotein	*p60*	413	818435…819676
Mbr-GS01GM000691	putative membrane protein P80	*p80*	722	819676…821844

Lipoproteins on the *Mycoplasma* surface play a crucial role in interactions between pathogen and eukaryotic cells, antigenic variation, and immunity evasion. Thus, they are responsible for *Mycoplasma* virulence (Bürki *et al.* 2015). Seven surface or membrane-associated lipoproteins (Table 1), including P60-like proteins and seven other lipoproteins, were found. P60 is regarded as a virulence factor of *M. hyopneumoniae* (Seymour *et al.* 2012). These lipoproteins may be virulence factors of *M. bovirhinis*.

Glycerol metabolism and its metabolic product H_2_O_2_ contributes to the virulence of *Mycoplasma* (Blötz and Stülke 2017; Hames *et al.* 2009). The *glpF-glpK-glpD* gene cluster was found in the *M. bovirhinis* genome (Table 1). The gene cluster *gtsABC* is an efficient active glycerol import system and is found in many *Mycoplasma* genomes, such as those in *M. mycoides* subsp. *mycoides* SC (Blötz and Stülke 2017), *M. pneumoniae* (Hames *et al.* 2009), and *M. capricolum* subsp. *capripneumoniae* (Mccp) (Chen *et al.* 2017), but no gene cluster *gtsABC* was identified in the GS01 genome.

Magnesium transporters MgtA and MgtE are considered virulence factors in some bacteria (Groisman *et al.* 2013). The potassium transporter TrkA is related to the virulence of *Salmonella* (Su *et al.* 2009). In the GS01 genome, three genes, namely, *mgtA* (Mbr-GS01GM000528), *mgtE* (Mbr-GS01GM000661), and *trkA* (Mbr-GS01GM000385), were predicted, and their encoding proteins may be related to the virulence of *M. bovirhinis*.

A 5ʹ-nucleotidase that utilizes host nucleotides and can enhance macrophage death is considered a virulence factor in *Streptococcus pyogenes* (Zheng *et al.* 2015). A 5ʹ-nucleotidase gene (Mbr-GS01GM000652) was identified in the genome and may be associated with the virulence of *M. bovirhinis*.

### Comparative and Evolutionary Analysis

**Figure 2:**
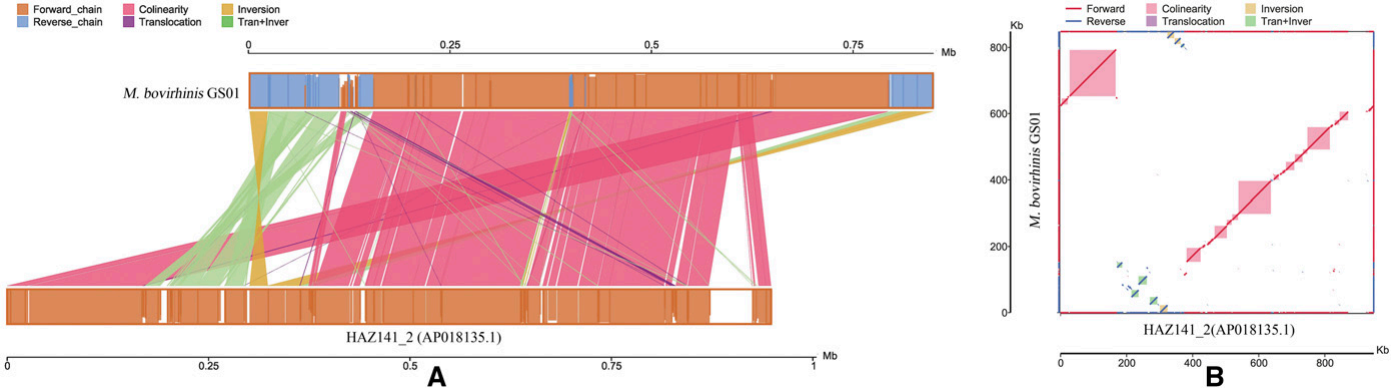
Collinearity analysis of the complete genome between *M. bovirhinis* strain GS01 and HAZ141_2. (a): Two genomes on parallel collinearity. The upper and lower shafts represent the sequenced and reference genomes, respectively. The orange box in the axes indicates the genomic forward strand, and the blue one corresponds to the reverse chain. The filled color in the box denotes alignment similarity, and the complete filling suggests 100% similarity. The color of the link graph between the upper and lower axes represents alignment type: pink, purple, yellow, and green denote collinear, translocation, inversion, and translocation+inversion, respectively. (b): Two-dimensional comparison between two genomes. The vertical and horizontal axes represent GS01 genome and reference HAZ141_2 genome, respectively. The blue line shows the reverse chain, and the red line corresponds to forward alignment. The pink and green modules indicate collinear and translocation+inversion between them, respectively.

The genome comparisons between *M. bovirhinis* GS01 and HAZ141_2 strains were conducted and are shown in Table S9 in File S1. The HAZ141_2 genome size is 948,039 bp, 100,054 bp longer than the GS01 genome. The overall sequence similarity of GS01 and HAZ141_2 was calculated to be 97.56%. GS01 has 707 protein-encoding genes, and HAZ141_2 was annotated to have 821 protein-encoding genes using the same genome annotation method as GS01. 16 and 38 pseudogenes were separately identified in GS01 and HAZ141_2 according to the annotation by the NCBI Prokaryotic Genome Annotation Pipeline on the GenBank database. The functional category in COG of *M. bovirhinis* GS01 and HAZ141_2 were compared and are shown in Table S3 in File S1. The collinearity analysis between GS01 and HAZ141_2 was also conducted. The origin of HAZ141_2 genome was 752 bp upstream of the RNase J family beta-CASP ribonuclease gene, while the *dnaA* gene was at position 1 for GS01 as majority *Mycoplasma*. Their genome structures did not have very high synteny (Figure 2), with 130 blocks in the comparison. Approximately 12.3-kb translocation + inversion was found at the beginning of genome GS01 and 60-kb inversion at the end and start. The genome size of GS01 was 100-kb smaller than that of HAZ141_2. Several insertions and deletions were observed, and the largest deletion was 53,407 bp long. The 53.4-kb deletion in GS01 relative to HAZ141_2 was sited from 871,457 to 924,505, correspond to the 53.5-kb insertion identified in HAZ141_2 by Hata *et al.* (2017). The corresponding segment encoded 47 proteins, mainly including phage-related proteins, hypothetical proteins, and others (Table S10 in File S1).

**Figure 3:**
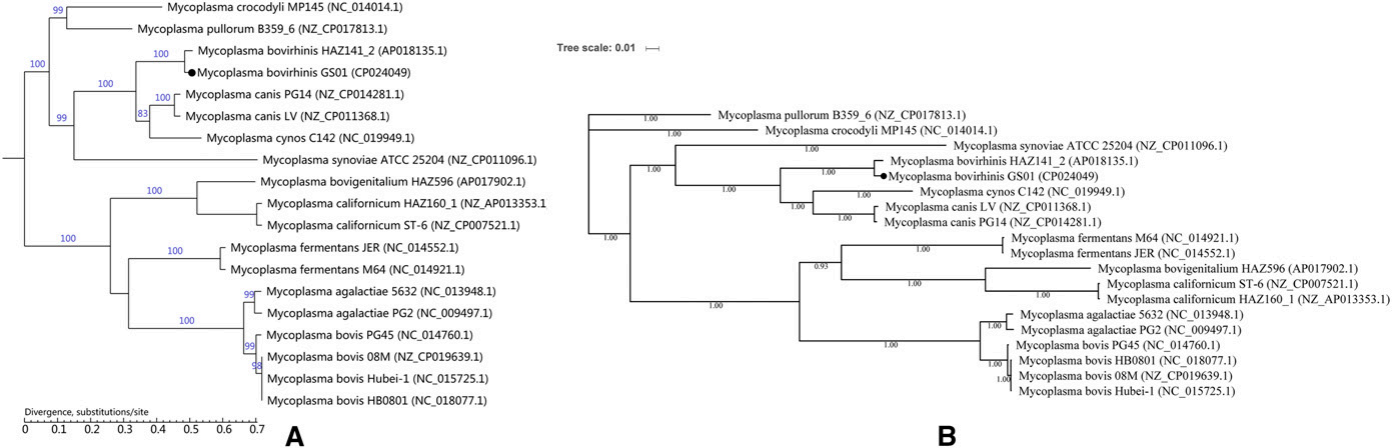
Phylogenetic trees of 14 single-copy core genes of 19 selected *Mycoplasma* strains. (a): Phylogenetic tree was constructed using TreeBeST with the maximum likelihood model. Bootstrap replicates were 1000, and the number of bootstraps for each node is shown. The tree is displayed to scale, with branch lengths measured in the number of substitutions per site. *M. bovirhinis* strain GS01 is indicated by a black dot. (b): Phylogenetic tree was constructed using MrBayes with bayesian inference method. The numbers upon each node indicate bayesian posterior probabilities. The tree is displayed to scale, with branch lengths measured in the number of substitutions per site. *M. bovirhinis* strain GS01 is indicated by a black dot.

The core genes between *M. bovirhinis* GS01 and 18 other *Mycoplasma* genomes were identified using CD-HIT, and 14 single-copy core genes were found (Table S11 in File S1). Phylogenetic trees were performed based on the single-copy core genes of 19 *Mycoplasma* strains using maximum likelihood and bayesian inference methods, and the topology of the phylogeny were consistent (Figure 3). According to the phylogenetic trees, the *M. bovirhinis* GS01 and HAZ141_2 strains have the nearest relationship as expected. *M. bovirhinis* was closely related with *M. canis*, followed by *M. cynos*, but was distant from *M. bovis*, which is also an important bovine respiratory and mastitis pathogen.

## Discussion

Enolase, which catalyzes the conversion of 2-phosphoglycerate (2-PGA) to phosphoenolpyruvate (PEP) during glycolysis and a reverse reaction during glycogen synthesis (Spring and Wold 1971), is a plasminogen-binding protein and is closely related to the adherence to the host cell in many mycoplasma species, such as *M. gallisepticum* (Chen *et al.* 2011), *M. bovis* (Song *et al.* 2012) and *M. synoviae* (Bao *et al.* 2014). An enolase was identified in the GS01 genome, also in HAZ141_2, and may be considered an adherence and virulence factor in *M. bovirhinis*, but its role in this *Mycoplasma* species needs further investigations.

Glycerol metabolism and H_2_O_2_ production influence *Mycoplasma* virulence (Hames *et al.* 2009; Vilei and Frey 2001). Glycerol uptake usually occurs through an efficient active glycerol import system GtsABC, and possibly a bypass pathway via the glycerol facilitator factor GlpF. In the GS01 genome, the *gtsABC* gene cluster were absent. Glycerol metabolism may be supported by the *glpF-glpK-glpD* gene cluster, but need verification. In HAZ141_2, *gtsABC* and *glpF-glpK-glpD* gene clusters existed, indicating that glycerol metabolism may vary among *M. bovirhinis* strains.

In addition to 16 putative virulence genes predicted in this study, ClpB (Chastanet *et al.* 2004), ClpC (Nair *et al.* 2000), hemolysins (Goebel *et al.* 1988), PDH enzyme complex (Gates *et al.* 2008; Gründel *et al.* 2015), and lipoate-protein ligase LplA (O’Riordan *et al.* 2003) are regarded as relevant virulence factors in bacteria. These genes are not found in *M. bovirhinis* GS01 and HAZ141_2 genomes, but were present in genomes of *M. bovis* (Li *et al.* 2011) and Mccp (Chen *et al.* 2017), important contagious pleuropneumonia pathogens for cattle and goats, respectively. The absence of these crucial virulence factors may be associated with the relatively lower pathogenicity of *M. bovirhinis* than that of *M. bovis* as expected.

Signal peptidase I (SPase I) is for general protein secretion, whereas signal peptidase II, also called lipoprotein signal peptidase (LspA), functions by releasing signal peptides from bacterial prolipoproteins. Both signal peptidase I and II genes are found in the *M. bovirhinis* GS01 and HAZ141_2 genomes, *M. synoviae* (Vasconcelos *et al.* 2005), *M. conjunctivae* (Calderon-Copete *et al.* 2009), and *M. hyopneumoniae* (Moitinho-Silva *et al.* 2012). For the genomes of other *Mycoplasma* species, such as *M. bovis* and Mccp, only the signal peptidase II gene has been identified (Li *et al.* 2011; Chen *et al.* 2017). This result indicates that the mechanism of protein secretion in *M. bovirhinis* may be the same as that in *M. synoviae*, *M. conjunctivae*, and *M. hyopneumoniae*, but different from that in Mccp and *M. bovis*.

Two genomic sequences of *M. bovirhinis* have now been determined. GS01 had the nearest relationship with HAZ141_2 based on the phylogenetic tree and comparative analysis. Moderate genomic synteny and a large-scale deletion in GS01 relative to HAZ141_2 indicated remarkable genome variations of *M. bovirhinis. M. bovirhinis* was most genetically close to *M. canis*, which is usually considered a commensal or opportunistic cofactor in respiratory or urogenital tract diseases of dogs (Ulgen *et al.* 2006) and can be recovered from pneumonic calves (ter Laak *et al.* 1993). The genetics and phylogenetic of *M. bovirhinis* will be revealed more clearly with the aid of more strains sequenced in the future.

## 

## Supplementary Material

Supplemental material is available online at www.g3journal.org/lookup/suppl/doi:10.1534/g3.118.200018/-/DC1.
