# Supplementary material for: Genome-Wide Analysis of Mycoplasma bovirhinis GS01 Reveals Potential Virulence Factors and Phylogenetic Relationships
Source: G3 (Bethesda). 2018 Mar 30;8(5):1417–24. doi: 10.1534/g3.118.200018 (PMC5940136; doi:10.1534/g3.118.200018)
Supplement: Supplementary file 1 [file 1417FileS1.zip › Supplementary Materials/Table S1 Prediction of the genome components of M. bovirhinis GS01.doc]

**Table S1 Prediction of the genome components of *M. bovirhinis* GS01**

| Description | Results |
| --- | --- |
| Genome size (bp) | 847,985 |
| Sequencing depth | 1026× |
| Coverage | 650× |
| GC content | 27.57% |
| Protein-coding genes (excluding pseudogenes) | 707 |
| Protein-coding gene length (bp) | 757,599 |
| Gene/Genome (%) | 89.34% |
| GC content in gene region | 27.96% |
| Gene average length (bp) | 1,072 |
| Intergenic region length (bp) | 90,386 |
| GC content in intergenic region | 24.30% |
| Intergenic length/Genome (%) | 10.66% |
| tRNA number | 31 |
| tRNA average length (bp) | 78 |
| rRNA (by de novo prediction) | 8 |
| 5S rRNA (by de novo prediction) | 2 |
| 16S rRNA (by de novo prediction) | 3 |
| 23S rRNA (by de novo prediction) | 3 |
| snRNA | 0 |
| Genomics islands number | 3 |
| Genomics islands average length (bp) | 10,847 |
