# Supplementary material for: Genome-Wide Analysis of Mycoplasma bovirhinis GS01 Reveals Potential Virulence Factors and Phylogenetic Relationships
Source: G3 (Bethesda). 2018 Mar 30;8(5):1417–24. doi: 10.1534/g3.118.200018 (PMC5940136; doi:10.1534/g3.118.200018)
Supplement: Supplementary file 1 [file 1417FileS1.zip › Supplementary Materials/Table S10 The genes of the 53.4-kb deletion in GS01 relative to HAZ141_2.doc]

**Table S10 The genes of the 53.4-kb deletion in GS01 relative to HAZ141_2**

| Locus^a^ | Product | Protein length (aa) | Position | Forward (+)/  Reverse(-) chain |
| --- | --- | --- | --- | --- |
| MBVR141_0923 | transposase | 1536 | 871407…872939 | + |
| MBVR141_0925 | pyrrolidone-carboxylate peptidase | 303 | 873435…873737 | - |
| MBVR141_0926 | hypothetical protein | 489 | 873762…874250 | + |
| MBVR141_0927 | hypothetical protein | 675 | 874234…874908 | + |
| MBVR141_0929 | aminoglycoside phosphotransferase APH(3') | 795 | 875016…875810 | - |
| MBVR141_0931 | streptothricin N-acetyltransferase Sat4 | 543 | 875903…876445 | - |
| MBVR141_0932 | streptomycin adenylyltransferase | 687 | 876376…877062 | - |
| MBVR141_0934 | hypothetical protein | 1647 | 877342…878988 | - |
| MBVR141_0937 | recombinase family protein | 1323 | 878915…880237 | - |
| MBVR141_0940 | helix-turn-helix domain-containing protein | 405 | 880447…880851 | - |
| MBVR141_0942 | hypothetical protein | 381 | 880944…881324 | - |
| MBVR141_0944 | hypothetical protein | 3384 | 881994…885377 | - |
| MBVR141_0950 | phage tail component | 792 | 885302…886093 | - |
| MBVR141_0951 | phage tail protein | 2700 | 886000…888699 | - |
| MBVR141_0955 | hypothetical protein | 384 | 888908…889291 | - |
| MBVR141_0958 | phi13 family phage major tail protein | 603 | 889291…889893 | - |
| MBVR141_0959 | hypothetical protein | 390 | 889899…890288 | - |
| MBVR141_0960 | hypothetical protein | 423 | 890228…890650 | - |
| MBVR141_0962 | phage head-tail adapter protein | 402 | 890593…890994 | - |
| MBVR141_0964 | hypothetical protein | 327 | 890982…891308 | - |
| MBVR141_0966 | phage major capsid protein | 1200 | 891320…892519 | - |
| MBVR141_0968 | peptidase | 780 | 892534…893313 | - |
| MBVR141_0970 | phage portal protein | 1317 | 893186…894502 | - |
| MBVR141_0973 | terminase | 1662 | 894514…896175 | - |
| MBVR141_0977 | methyltransferase | 384 | 896189…896572 | - |
| MBVR141_0979 | DNA modification methylase | 1362 | 896836…898197 | - |
| MBVR141_0983 | hypothetical protein | 666 | 899075…899740 | - |
| MBVR141_0986 | hypothetical protein | 396 | 899747…900142 | - |
| MBVR141_0988 | hypothetical protein | 327 | 900123…900449 | - |
| MBVR141_0990 | S-adenosylmethionine synthetase | 897 | 900465…901361 | - |
| MBVR141_0992 | terminase | 546 | 901253…901798 | - |
| MBVR141_0994 | HNH endonuclease | 351 | 901919…902269 | - |
| MBVR141_0995 | hypothetical protein | 447 | 902331…902777 | - |
| MBVR141_0999 | crossover junction endodeoxyribonuclease RusA | 462 | 903019…903480 | - |
| MBVR141_1000 | DNA primase | 2025 | 903705…905729 | - |
| MBVR141_1004 | DEAD/DEAH box helicase | 1587 | 905746…907332 | - |
| MBVR141_1011 | hypothetical protein | 468 | 907339…907806 | - |
| MBVR141_1013 | hypothetical protein | 963 | 907809…908771 | - |
| MBVR141_1018 | hypothetical protein | 1266 | 908765…910030 | - |
| MBVR141_1023 | RNA polymerase subunit sigma | 459 | 910190…910648 | - |
| MBVR141_1025 | transcriptional regulator | 1935 | 911244…913178 | - |
| MBVR141_1027 | type I restriction enzyme, R subunit | 2916 | 913199…916114 | - |
| MBVR141_1033 | Site-specific recombinase XerD | 975 | 916639…917613 | - |
| MBVR141_1035 | type I restriction enzyme, S subunit | 1050 | 917665…918714 | - |
| MBVR141_1037 | type I restriction enzyme M protein | 1545 | 918663…920207 | - |
| MBVR141_1039 | restriction endonuclease subunit M | 2457 | 920197…922653 | - |
| MBVR141_1042 | restriction endonuclease subunit S | 1566 | 922611…924176 | - |

^a^ means the information was retrieved from NCBI database (accession No. AP018135.1)
