# Supplementary material for: Genome-Wide Analysis of Mycoplasma bovirhinis GS01 Reveals Potential Virulence Factors and Phylogenetic Relationships
Source: G3 (Bethesda). 2018 Mar 30;8(5):1417–24. doi: 10.1534/g3.118.200018 (PMC5940136; doi:10.1534/g3.118.200018)
Supplement: Supplementary file 1 [file 1417FileS1.zip › Supplementary Materials/Table S11 List of 14 single-copy core genes of 19 selected Mycoplasma strains.doc]

**Table S11 List of 14 single-copy core genes of 19 selected *Mycoplasma* strains**

| Locus^a^ | Product^a^ | Protein length (aa)^a^ | Position^a^ | Forward (+)/  Reverse(-) chain^a^ |
| --- | --- | --- | --- | --- |
| Mbr-GS01GM000046 | elongation factor Tu | 395 | 40880:42067 | - |
| Mbr-GS01GM000221 | 50S ribosomal protein L3 | 262 | 242847:243635 | + |
| Mbr-GS01GM000226 | 50S ribosomal protein L22 | 114 | 246135:246479 | + |
| Mbr-GS01GM000230 | 30S ribosomal protein S17 | 86 | 247742:248002 | + |
| Mbr-GS01GM000231 | 50S ribosomal protein L14 | 121 | 248002:248367 | + |
| Mbr-GS01GM000235 | 30S ribosomal protein S8 | 131 | 249449:249844 | + |
| Mbr-GS01GM000236 | 50S ribosomal protein L6 | 180 | 249853:250395 | + |
| Mbr-GS01GM000237 | 50S ribosomal protein L18 | 116 | 250417:250767 | + |
| Mbr-GS01GM000238 | 30S ribosomal protein S5 | 226 | 250770:251450 | + |
| Mbr-GS01GM000302 | F0F1 ATP synthase subunit beta | 467 | 340543:341946 | + |
| Mbr-GS01GM000324 | purine nucleoside phosphorylase | 234 | 362760:363464 | + |
| Mbr-GS01GM000369 | bifunctional protein FolD | 277 | 412345:413178 | + |
| Mbr-GS01GM000405 | ribosomal RNA large subunit methyltransferase H | 144 | 465965:466399 | + |
| Mbr-GS01GM000627 | translation initiation factor IF-2 | 601 | 737640:739445 | + |

^a^ The information was corresponding to *M. bovirhinis* GS01. The listed 14 single-copy core genes can be found in 19/19 selected *Mycoplasma* strains.
