# Supplementary material for: Genome-Wide Analysis of Mycoplasma bovirhinis GS01 Reveals Potential Virulence Factors and Phylogenetic Relationships
Source: G3 (Bethesda). 2018 Mar 30;8(5):1417–24. doi: 10.1534/g3.118.200018 (PMC5940136; doi:10.1534/g3.118.200018)
Supplement: Supplementary file 1 [file 1417FileS1.zip › Supplementary Materials/Table S2 The statistical results of repetitive sequences of M. bovirhinis GS01.doc]

**Table S2 The statistical results of repetitive sequences of *M. bovirhinis* GS01**

| Type | | Number | Repeat Size (bp) | In Genome (%) | Average length (bp) |
| --- | --- | --- | --- | --- | --- |
| Interspersed nuclear elements | LTR | 38 | 2,957 | 0.3487 | 80 |
|  | DNA | 11 | 784 | 0.0925 | 71 |
|  | LINE | 12 | 894 | 0.1054 | 74 |
|  | SINE | 3 | 225 | 0.0265 | 75 |
|  | RC | 3 | 225 | 0.0265 | 75 |
|  | Unknown | 1 | 72 | 0.0085 | 72 |
|  | Total | 68 | 4,887 | 0.5763 | 77 |
| Tandem repeat | TR | 67 | 2~192 | 0.5456 | 69 |
|  | Minisatellite DNA | 51 | 12~60 | 0.4551 | 76 |
|  | Microsatellite DNA | 7 | 2~6 | 0.0501 | 61 |

Note that the LTR means long terminal repeat, DNA means DNA transposons, RC represents rolling circle, LINE represents long interspersed nuclear elements, SINE represents short interspersed nuclear elements, and TR means tandem repeat.
