# Supplementary material for: Genome-Wide Analysis of Mycoplasma bovirhinis GS01 Reveals Potential Virulence Factors and Phylogenetic Relationships
Source: G3 (Bethesda). 2018 Mar 30;8(5):1417–24. doi: 10.1534/g3.118.200018 (PMC5940136; doi:10.1534/g3.118.200018)
Supplement: Supplementary file 1 [file 1417FileS1.zip › Supplementary Materials/Table S3 Functional category in COG of M. bovirhinis.docx]

**Table S3 Functional category in COG of *M. bovirhinis***

| Code | Functional category | GS01 | HAZ141_2 | Common |
| --- | --- | --- | --- | --- |
| C | Energy production and conversion | 11 | 10 | 10 |
| D | Cell cycle control, cell division, chromosome partitioning | 2 | 3 | 2 |
| E | Amino acid transport and metabolism | 15 | 15 | 15 |
| F | Nucleotide transport and metabolism | 17 | 17 | 17 |
| G | Carbohydrate transport and metabolism | 27 | 28 | 27 |
| H | Coenzyme transport and metabolism | 8 | 11 | 8 |
| I | Lipid transport and metabolism | 3 | 2 | 2 |
| J | Translation, ribosomal structure and biogenesis | 115 | 120 | 112 |
| K | Transcription | 7 | 10 | 7 |
| L | Replication, recombination and repair | 19 | 27 | 19 |
| M | Cell wall/membrane/envelope biogenesis | 1 | 1 | 1 |
| O | Posttranslational modification, protein turnover, chaperones | 8 | 12 | 8 |
| P | Inorganic ion transport and metabolism | 10 | 9 | 9 |
| Q | Secondary metabolites biosynthesis, transport and catabolism | 1 | 1 | 1 |
| R | General function prediction only | 12 | 14 | 12 |
| S | Function unknown | 1 | 1 | 1 |
| T | Signal transduction mechanisms | 3 | 2 | 2 |
| U | Intracellular trafficking, secretion, and vesicular transport | 4 | 4 | 4 |
| V | Defense mechanisms | 12 | 15 | 11 |
| X | Mobilome: prophages, transposons | 6 | 8 | 2 |
| Total | - | 282 | 310 | 270 |
