# Supplementary material for: Genome-Wide Analysis of Mycoplasma bovirhinis GS01 Reveals Potential Virulence Factors and Phylogenetic Relationships
Source: G3 (Bethesda). 2018 Mar 30;8(5):1417–24. doi: 10.1534/g3.118.200018 (PMC5940136; doi:10.1534/g3.118.200018)
Supplement: Supplementary file 1 [file 1417FileS1.zip › Supplementary Materials/Table S4 Genes predicted involving in transporter system of M. bovirhinis GS01.doc]

**Table S4 Genes predicted involving in transporter system of *M. bovirhinis* GS01**

| Locus | Product | Gene | Gene length (bp) | Protein length (aa) | Position |
| --- | --- | --- | --- | --- | --- |
| ABC transporters | | | | | |
| Mbr-GS01GM000151 | maltose/maltodextrin transporter ATP-binding protein | *malK* | 1224 | 407 | 167528…168751 |
| Mbr-GS01GM000152 | maltodextrin ABC transporter, permease MalD | *malG* | 963 | 320 | 168758…169720 |
| Mbr-GS01GM000153 | maltose/maltodextrin ABC transporter, permease protein | *malF* | 3063 | 1020 | 169723…172785 |
| Mbr-GS01GM000189 | cobalt/nickel ABC transporter ATP-binding protein | *ecfA1* | 816 | 271 | 210899…211714 |
| Mbr-GS01GM000190 | cobalt/nickel ABC transporter ATP-binding protein | *ecfA2* | 888 | 295 | 211690…212577 |
| Mbr-GS01GM000191 | cobalt/nickel transport system permease protein | *ecfT* | 1056 | 351 | 212579…213634 |
| Mbr-GS01GM000277 | spermidine/putrescine transport system ATP-binding protein | *potA* | 1386 | 461 | 304561…305946 |
| Mbr-GS01GM000278 | spermidine/putrescine ABC transporter permease | *potB* | 834 | 277 | 305930…306763 |
| Mbr-GS01GM000279 | spermidine/putrescine transport system permease | *potC* | 786 | 261 | 306756…307541 |
| Mbr-GS01GM000283 | putative oligopeptide ABC transporter solute binding protein | *oppA* | 3021 | 1006 | 317934…320954 |
| Mbr-GS01GM000284 | dipeptide/oligopeptide ABC transporter permease | *oppB* | 1038 | 345 | 320957…321994 |
| Mbr-GS01GM000285 | oligopeptide transport system permease | - | 1041 | 346 | 321987…323027 |
| Mbr-GS01GM000286 | dipeptide/oligopeptide ABC transporter ATP-binding protein | *oppD* | 2244 | 747 | 323039…325282 |
| Mbr-GS01GM000287 | dipeptide/oligopeptide ABC transporter ATP-binding protein | *amiF* | 1332 | 443 | 325263…326594 |
| Mbr-GS01GM000305 | ABC transporter ATP-binding protein | - | 2001 | 666 | 343175…345175 |
| Mbr-GS01GM000335 | sugar ABC transporter ATP-binding protein | - | 2091 | 696 | 376871…378961 |
| Mbr-GS01GM000336 | sugar ABC transporter, permease protein | - | 1047 | 348 | 378942…379988 |
| Mbr-GS01GM000337 | sugar ABC transporter permease protein | - | 1041 | 346 | 379936…380976 |
| Mbr-GS01GM000396 | multidrug-like ABC transporter ATP-binding protein | - | 711 | 236 | 450882…451592 |
| Mbr-GS01GM000397 | putative ABC transporter protein | - | 1890 | 629 | 451592…453481 |
| Mbr-GS01GM000460 | multidrug ABC transporter ATPase | - | 1755 | 584 | 540637…542391 |
| Mbr-GS01GM000461 | ABC transporter superfamily protein | - | 1845 | 614 | 542394…544238 |
| Mbr-GS01GM000536 | oligopeptide ABC transporter, ATP-binding protein | *oppF* | 2445 | 814 | 628525…630969 |
| Mbr-GS01GM000537 | oligopeptide ABC transporter ATP-binding protein | *oppD* | 1053 | 350 | 630962…632014 |
| Mbr-GS01GM000538 | peptide/nickel transport system permease protein | - | 1320 | 439 | 632027…633346 |
| Mbr-GS01GM000539 | oligopeptide ABC transporter, permease protein | *oppB* | 1182 | 393 | 633336…634517 |
| Mbr-GS01GM000544 | heme ABC transporter ATP-binding protein | *ykpA* | 1647 | 548 | 642726…644372 |
| Mbr-GS01GM000562 | ABC transporter ATP-binding protein | *-* | 1983 | 660 | 664128…666110 |
| Mbr-GS01GM000581 | ABC transporter, permease protein | - | 1233 | 410 | 696066…697298 |
| Mbr-GS01GM000648 | sugar ABC transporter permease protein | - | 948 | 315 | 759446…760393 |
| Mbr-GS01GM000649 | sugar ABC transporter permease protein | - | 1614 | 537 | 760395…762008 |
| Mbr-GS01GM000650 | unspecified sugar ABC transport ATP-binding protein | *yufO* | 2265 | 754 | 762008…764272 |
| Mbr-GS01GM000651 | sugar ABC transporter substrate-binding protein | - | 1347 | 448 | 764368…765714 |
| Mbr-GS01GM000653 | sugar ABC transporter substrate-binding protein | - | 1938 | 645 | 768496…770433 |
| Mbr-GS01GM000654 | ABC transporter, ATP-binding protein | - | 1818 | 605 | 770446…772263 |
| Mbr-GS01GM000668 | phosphate/phosphonate ABC transporter, ATP-binding protein | *phnC* | 723 | 240 | 788942…789664 |
| Mbr-GS01GM000669 | ABC transport system permease protein | - | 1617 | 538 | 789657…791273 |
| Mbr-GS01GM000702 | ABC transporter, ATP-binding protein | *vraD* | 963 | 320 | 832856…833818 |
| Mbr-GS01GM000703 | ABC transporter permease protein | - | 7965 | 2654 | 833820…841784 |
| Mbr-GS01GM000063 | excinuclease ABC subunit A | *uvrA* | 2853 | 950 | 57026…59878 |
| Mbr-GS01GM000240 | excinuclease ABC subunit C | *uvrC* | 1809 | 602 | 252502…254310 |
| Mbr-GS01GM000672 | excinuclease ABC subunit B | *uvrB* | 1989 | 662 | 793854…795842 |
| PTS system | | | | | |
| Mbr-GS01GM000174 | PTS system fructose-specific EIIABC component | *fruA* | 2070 | 689 | 199242…201311 |
| Mbr-GS01GM000356 | PTS system lichenan-specific IIA component LicA | *licA* | 747 | 248 | 401821…402567 |
| Mbr-GS01GM000448 | PTS system phosphocarrier protein HPr | *ptsH* | 264 | 87 | 524655…524918 |
| Mbr-GS01GM000579 | phosphoenolpyruvate-protein phosphotransferase | *ptsI* | 1704 | 567 | 693983…695686 |
| Mbr-GS01GM000585 | PTS system glucose-specific IIABC component | *gamP* | 2319 | 772 | 701993…704311 |
| Mbr-GS01GM000594 | PTS system glucose-specific IIABC component | - | 516 | 171 | 711637…712152 |
| Others | | | | | |
| Mbr-GS01GM000384 | potassium transporter KtrB | - | 1863 | 620 | 429531…431393 |
| Mbr-GS01GM000385 | potassium transporter TrkA | *trkA* | 672 | 223 | 431400…432071 |
| Mbr-GS01GM000478 | metal cation transporter, ZIP family protein | - | 1035 | 344 | 565034…566068 |
| Mbr-GS01GM000491 | glycerol uptake facilitator protein | *glpF* | 747 | 248 | 581842…582588 |
| Mbr-GS01GM000528 | magnesium transporter | *mgtE* | 1575 | 524 | 621734…623308 |
| Mbr-GS01GM000661 | magnesium-transporting ATPase (P-type) | *mgtA* | 2715 | 904 | 779505…782219 |
| Mbr-GS01GM000662 | chromate ion transporter | *chrA* | 657 | 218 | 782234…782890 |
| Mbr-GS01GM000663 | chromate ion transporter | *chrA* | 570 | 189 | 782894…783463 |
