# Supplementary material for: Genome-Wide Analysis of Mycoplasma bovirhinis GS01 Reveals Potential Virulence Factors and Phylogenetic Relationships
Source: G3 (Bethesda). 2018 Mar 30;8(5):1417–24. doi: 10.1534/g3.118.200018 (PMC5940136; doi:10.1534/g3.118.200018)
Supplement: Supplementary file 1 [file 1417FileS1.zip › Supplementary Materials/Table S5 The predicted genes involving in metabolism in the GS01 genome.doc]

**Table S5 The predicted genes involving in metabolism in the GS01 genome**

| Locus | Product | Gene | Class | Protein length (aa) | Position |
| --- | --- | --- | --- | --- | --- |
| Mbr-GS01GM000027 | glycerol-3-phosphate acyltransferase | *plsY* | lipid metabolism | 240 | 20405…21127 |
| Mbr-GS01GM000028 | uridylate kinase | *pyrH* | nucleotide metabolism | 239 | 21198…21917 |
| Mbr-GS01GM000044 | uracil phosphoribosyltransferase | *upp* | nucleotide metabolism | 206 | 37620…38240 |
| Mbr-GS01GM000049 | putative phosphoketolase | - | carbohydrate metabolism | 806 | 43156…45576 |
| Mbr-GS01GM000055 | thymidylate kinase | *tmk* | nucleotide metabolism | 215 | 50327…50974 |
| Mbr-GS01GM000058 | DNA polymerase III subunit gamma/tau | *dnaX* | nucleotide metabolism; | 617 | 51895…53748 |
| Mbr-GS01GM000061 | cytidylate kinase | *cmk* | nucleotide metabolism | 229 | 55394…56083 |
| Mbr-GS01GM000066 | thioredoxin-disulfide reductase | *trxB* | nucleotide metabolism | 310 | 61855…62787 |
| Mbr-GS01GM000091 | asparagine synthetase AsnA | *asnA* | amino acid metabolism | 327 | 105895…106878 |
| Mbr-GS01GM000130 | leucyl aminopeptidase | *pepA* | metabolism of other amino acids | 458 | 144024…145400 |
| Mbr-GS01GM000135 | glycerophosphoryl diester phosphodiesterase | *glpQ* | lipid metabolism | 245 | 149084…149821 |
| Mbr-GS01GM000144 | beta-phosphoglucomutase or related phosphatase, HAD superfamily | *pgmB* | carbohydrate metabolism | 226 | 157163…157843 |
| Mbr-GS01GM000146 | sucrase-isomaltase | *malZ* | carbohydrate metabolism | 610 | 160253…162085 |
| Mbr-GS01GM000157 | alpha-amylase | *malS* | carbohydrate metabolism | 638 | 176276…178192 |
| Mbr-GS01GM000158 | proline iminopeptidase | *pip* | amino acid metabolism | 335 | 178276…179283 |
| Mbr-GS01GM000165 | eucyl aminopeptidase | *pepA* | metabolism of other amino acids | 462 | 185057…186445 |
| Mbr-GS01GM000167 | alpha-amylase | *malS* | carbohydrate metabolism | 672 | 189890…191908 |
| Mbr-GS01GM000173 | nicotinic acid phosphoribosyltransferase | *pncB* | metabolism of cofactors and vitamins | 334 | 198091…199095 |
| Mbr-GS01GM000174 | PTS system, fructose-specific IIA component | *fruB* | carbohydrate metabolism | 689 | 199242…201311 |
| Mbr-GS01GM000175 | 1-phosphofructokinase | *fruK* | carbohydrate metabolism | 303 | 201314…202225 |
| Mbr-GS01GM000185 | DNA (cytosine-5-)-methyltransferase | *dcm* | amino acid metabolism | 322 | 207943…208911 |
| Mbr-GS01GM000195 | glycerol-3-phosphate acyltransferase PlsX | *plsX* | lipid metabolism | 327 | 220379…221362 |
| Mbr-GS01GM000208 | nitrogen fixation protein NifS | *sufS* | metabolism of other amino acids | 385 | 230636…231793 |
| Mbr-GS01GM000211 | nicotinate-nucleotide adenylyltransferase | *nadD* | metabolism of cofactors and vitamins | 357 | 233448…234521 |
| Mbr-GS01GM000214 | glucose-6-phosphate isomerase | *pgi* | carbohydrate metabolism | 428 | 235828…237114 |
| Mbr-GS01GM000215 | glutamyl-tRNA synthetase | *gltX* | metabolism of cofactors and vitamins | 466 | 237179…238579 |
| Mbr-GS01GM000250 | cytidine deaminase | *CDA* | nucleotide metabolism | 130 | 266511…266903 |
| Mbr-GS01GM000263 | fructose-bisphosphate aldolase | *fba* | carbohydrate metabolism | 288 | 290421…291287 |
| Mbr-GS01GM000269 | phosphopentomutase | *deoB* | carbohydrate metabolism | 395 | 296050…297237 |
| Mbr-GS01GM000292 | endoglucanase | - | carbohydrate metabolism | 363 | 331511…332602 |
| Mbr-GS01GM000296 | F-type H+-transporting ATPase subunit a | *atpB* | energy metabolism | 257 | 335879…336652 |
| Mbr-GS01GM000297 | F-type H+-transporting ATPase subunit c | *atpE* | energy metabolism | 101 | 336662…336967 |
| Mbr-GS01GM000298 | F-type H+-transporting ATPase subunit b | *atpF* | energy metabolism | 190 | 336979…337551 |
| Mbr-GS01GM000300 | F-type H+-transporting ATPase subunit alpha | *atpA* | energy metabolism | 527 | 338099…339682 |
| Mbr-GS01GM000301 | F-type H+-transporting ATPase subunit gamma | *atpG* | energy metabolism | 284 | 339675…340529 |
| Mbr-GS01GM000302 | F-type H+-transporting ATPase subunit beta | *atpD* | energy metabolism | 467 | 340543…341946 |
| Mbr-GS01GM000303 | F-type H+-transporting ATPase subunit epsilon | *atpC* | energy metabolism | 136 | 341946…342356 |
| Mbr-GS01GM000324 | purine-nucleoside phosphorylase | *deoD* | nucleotide metabolism | 234 | 362760…363464 |
| Mbr-GS01GM000325 | pyrimidine-nucleoside phosphorylase | *pdp* | nucleotide metabolism | 431 | 363469…364764 |
| Mbr-GS01GM000326 | deoxyribose-phosphate aldolase | *deoC* | carbohydrate metabolism | 220 | 364773…365435 |
| Mbr-GS01GM000331 | methionyl-tRNA synthetase | *metG* | metabolism of other amino acids | 514 | 370612…372156 |
| Mbr-GS01GM000345 | glucose-6-phosphate isomerase | *pgi* | carbohydrate metabolism | 429 | 389214…390503 |
| Mbr-GS01GM000346 | thymidine kinase | *tdk* | nucleotide metabolism | 192 | 390553…391131 |
| Mbr-GS01GM000347 | deoxyguanosine kinase | *dgk* | nucleotide metabolism | 220 | 391322…391984 |
| Mbr-GS01GM000348 | deoxyguanosine kinase | *dgk* | nucleotide metabolism | 225 | 391988…392665 |
| Mbr-GS01GM000364 | 1-acyl-sn-glycerol-3-phosphate acyltransferase | *plsC* | lipid metabolism | 243 | 406980…407711 |
| Mbr-GS01GM000365 | holo-[acyl-carrier protein] synthase | *acpS* | metabolism of cofactors and vitamins | 105 | 407753…408070 |
| Mbr-GS01GM000369 | Bifunctional protein FolD | *folD* | metabolism of cofactors and vitamins | 277 | 412345…413178 |
| Mbr-GS01GM000370 | 6-phosphofructokinase | *pfk* | carbohydrate metabolism | 325 | 413192…414169 |
| Mbr-GS01GM000382 | endoglucanase | - | carbohydrate metabolism | 365 | 428094…429191 |
| Mbr-GS01GM000406 | ubiquinone/menaquinone biosynthesis C-methylase UbiE | *UbiE* | metabolism of cofactors and vitamins | 240 | 466420…467142 |
| Mbr-GS01GM000408 | DNA-directed RNA polymerase subunit beta | *rpoB* | nucleotide metabolism | 1207 | 469093…472716 |
| Mbr-GS01GM000409 | DNA-directed RNA polymerase subunit beta' | *rpoC* | nucleotide metabolism | 1476 | 472709…477139 |
| Mbr-GS01GM000414 | D-lactate dehydrogenase | *ldhA* | carbohydrate metabolism | 345 | 485354…486391 |
| Mbr-GS01GM000435 | glyceraldehyde-3-phosphate dehydrogenase | *gapA* | carbohydrate metabolism | 333 | 509054…510055 |
| Mbr-GS01GM000436 | methionyl-tRNA formyltransferase | *fmt* | metabolism of cofactors and vitamins | 285 | 510215…511072 |
| Mbr-GS01GM000440 | DNA polymerase I | *polA* | nucleotide metabolism | 297 | 513539…514432 |
| Mbr-GS01GM000441 | DNA polymerase III subunit alpha | *dnaE* | nucleotide metabolism | 977 | 514422…517355 |
| Mbr-GS01GM000447 | phosphoglycerate kinase | *pgk* | carbohydrate metabolism | 395 | 523405…524592 |
| Mbr-GS01GM000449 | cardiolipin synthase | *cls* | lipid metabolism | 494 | 524984…526468 |
| Mbr-GS01GM000466 | F-type H+-transporting ATPase subunit beta | *atpD* | energy metabolism | 461 | 550340…551725 |
| Mbr-GS01GM000467 | F-type H+-transporting ATPase subunit alpha | *atpA* | energy metabolism | 509 | 551725…553254 |
| Mbr-GS01GM000476 | NAD+ synthetase | *nadE* | metabolism of cofactors and vitamins | 263 | 563499…564290 |
| Mbr-GS01GM000486 | phosphoglycerate mutase | *gpm* | carbohydrate metabolism | 501 | 576212…577717 |
| Mbr-GS01GM000489 | dihydroxyacetone kinase, L subunit | *dhaL* | lipid metabolism | 203 | 580175…580786 |
| Mbr-GS01GM000490 | dihydroxyacetone kinase subunit DhaK | *dhaK* | lipid metabolism | 327 | 580777…581760 |
| Mbr-GS01GM000492 | glycerol kinase | *glpK* | lipid metabolism | 505 | 582597…584114 |
| Mbr-GS01GM000493 | glycerol-3-phosphate dehydrogenase | *glpD* | lipid metabolism | 384 | 584124…585278 |
| Mbr-GS01GM000507 | PTS system, sucrose-specific IIB component | *scrA* | carbohydrate metabolism | 522 | 597723…599291 |
| Mbr-GS01GM000512 | triosephosphate isomerase (TIM） | *tpiA* | carbohydrate metabolism | 244 | 605753…606487 |
| Mbr-GS01GM000520 | glycine hydroxymethyltransferase | *glyA* | amino acid metabolism | 422 | 614038…615306 |
| Mbr-GS01GM000525 | CDP-diacylglycerol--glycerol-3-phosphate 3-phosphatidyltransferase | *pgsA* | lipid metabolism | 221 | 619879…620544 |
| Mbr-GS01GM000546 | inorganic pyrophosphatase | *ppa* | energy metabolism | 116 | 645098…645448 |
| Mbr-GS01GM000552 | inorganic pyrophosphatase | *ppa* | energy metabolism | 183 | 651631…652182 |
| Mbr-GS01GM000572 | S-adenosylmethionine synthetase | *metK* | amino acid metabolism | 379 | 687753…688892 |
| Mbr-GS01GM000574 | thiamine biosynthesis protein ThiI | *thiI* | metabolism of cofactors and vitamins | 386 | 690078…691238 |
| Mbr-GS01GM000577 | ribose 5-phosphate isomerase B | *rpiB* | carbohydrate metabolism | 148 | 692595…693041 |
| Mbr-GS01GM000585 | PTS system, D-glucosamine-specific IIA component | *gamP* | carbohydrate metabolism | 772 | 701993…704311 |
| Mbr-GS01GM000592 | glycerol-3-phosphate dehydrogenase (NAD(P)+) | *gpsA* | lipid metabolism | 330 | 708833…709825 |
| Mbr-GS01GM000596 | DNA-directed RNA polymerase subunit alpha | *rpoA* | nucleotide metabolism | 346 | 713684…714724 |
| Mbr-GS01GM000602 | adenylate kinase | *adk* | nucleotide metabolism | 212 | 716691…717329 |
| Mbr-GS01GM000621 | guanylate kinase | *gmk* | nucleotide metabolism | 207 | 732827…733450 |
| Mbr-GS01GM000623 | ribulose-phosphate 3-epimerase | *rpe* | carbohydrate metabolism | 223 | 734377…735048 |
| Mbr-GS01GM000628 | adenine phosphoribosyltransferase | *apt* | nucleotide metabolism | 169 | 739454…739963 |
| Mbr-GS01GM000631 | pantetheine-phosphate adenylyltransferase | *coaD* | metabolism of cofactors and vitamins | 144 | 741593…742027 |
| Mbr-GS01GM000632 | acetate kinase | *ackA* | metabolism of other amino acids | 395 | 742027…743214 |
| Mbr-GS01GM000638 | ribose-phosphate pyrophosphokinase | *prsA* | carbohydrate metabolism | 329 | 748651…749640 |
| Mbr-GS01GM000640 | pyruvate kinase | *pyk* | carbohydrate metabolism | 477 | 751942…753375 |
| Mbr-GS01GM000652 | 5'-nucleotidase | - | nucleotide metabolism | 707 | 765862…767985 |
| Mbr-GS01GM000686 | DNA polymerase III subunit alpha | polC | nucleotide metabolism | 1442 | 810455…814783 |
| Mbr-GS01GM000688 | enolase | *eno* | carbohydrate metabolism | 453 | 816615…817976 |
