# Supplementary material for: Genome-Wide Analysis of Mycoplasma bovirhinis GS01 Reveals Potential Virulence Factors and Phylogenetic Relationships
Source: G3 (Bethesda). 2018 Mar 30;8(5):1417–24. doi: 10.1534/g3.118.200018 (PMC5940136; doi:10.1534/g3.118.200018)
Supplement: Supplementary file 1 [file 1417FileS1.zip › Supplementary Materials/Table S6 Proteins involved in secretion system of M. bovirhinis GS01.doc]

**Table S6 Proteins involved in secretion system of *M. bovirhinis* GS01**

| Locus | Product | Gene | Gene length (bp) | Protein length (aa) | Position |
| --- | --- | --- | --- | --- | --- |
| Mbr-GS01GM000142 | fused signal recognition particle receptor FtsY | *ftsY* | 1047 | 348 | 155615…156661 |
| Mbr-GS01GM000205 | signal recognition particle subunit SRP54 | *ffh* | 1350 | 449 | 228074…229423 |
| Mbr-GS01GM000290 | molecular chaperone DnaK | *dnaK* | 1797 | 598 | 328593…330389 |
| Mbr-GS01GM000312 | competence protein ComEA | *comEA* | 501 | 166 | 350685…351185 |
| Mbr-GS01GM000320 | bifunctional preprotein translocase subunit SecD/SecF | *SecD* | 2556 | 851 | 357591…360146 |
| Mbr-GS01GM000328 | preprotein translocase subunit SecG | *secG* | 231 | 76 | 367394…367624 |
| Mbr-GS01GM000430 | preprotein translocase subunit SecE | *secE* | 213 | 70 | 504209…504421 |
| Mbr-GS01GM000542 | preprotein translocase subunit SecA | *secA* | 2520 | 839 | 639824…642343 |
| Mbr-GS01GM000557 | GTP-binding protein LepA | *lepA* | 1809 | 602 | 657209…659017 |
| Mbr-GS01GM000563 | signal peptidase I | *lepB* | 468 | 155 | 666116…666583 |
| Mbr-GS01GM000582 | signal peptidase II (lipoprotein signal peptidase) | *lspA* | 648 | 215 | 697787…698434 |
| Mbr-GS01GM000593 | trigger factor | - | 1314 | 437 | 709899…711212 |
| Mbr-GS01GM000603 | preprotein translocase subunit SecY | *secY* | 1431 | 476 | 717323…718753 |
| Mbr-GS01GM000694 | putative inner membrane protein translocase component YidC | *yidC* | 2115 | 704 | 824404…826518 |
