# Supplementary material for: Genome-Wide Analysis of Mycoplasma bovirhinis GS01 Reveals Potential Virulence Factors and Phylogenetic Relationships
Source: G3 (Bethesda). 2018 Mar 30;8(5):1417–24. doi: 10.1534/g3.118.200018 (PMC5940136; doi:10.1534/g3.118.200018)
Supplement: Supplementary file 1 [file 1417FileS1.zip › Supplementary Materials/Table S7 The effector proteins of T3SS in M. bovirhinis GS01 genome.doc]

**Table S7 The effector proteins of T3SS in *M. bovirhinis* GS01 genome**

| Locus | Product | Gene | Gene length (bp) | Protein length (aa) | Position |
| --- | --- | --- | --- | --- | --- |
| Mbr-GS01GM000024 | hypothetical protein | - | 894 | 297 | 17722…18615 |
| Mbr-GS01GM000038 | tRNA modification GTPase MnmE | *mnmE* | 1347 | 448 | 28756…30102 |
| Mbr-GS01GM000072 | 30S ribosomal protein S12 | *rpsL* | 411 | 136 | 71287…71697 |
| Mbr-GS01GM000083 | hypothetical protein | - | 1140 | 379 | 82451…83590 |
| Mbr-GS01GM000230 | 30S ribosomal protein S17 | *rpsQ* | 261 | 86 | 247742…248002 |
| Mbr-GS01GM000297 | ATP synthase subunit c | *atpE* | 306 | 101 | 336662…336967 |
| Mbr-GS01GM000298 | ATP synthase F0, B subunit | *atpF* | 573 | 190 | 336979…337551 |
| Mbr-GS01GM000403 | RNA-binding protein S1 | - | 2151 | 716 | 461953…464103 |
| Mbr-GS01GM000406 | type 11 methyltransferase | - | 723 | 240 | 466420…467142 |
| Mbr-GS01GM000420 | hypothetical protein BBOMB_1306, partial | - | 270 | 89 | 492055…492324 |
| Mbr-GS01GM000482 | hypothetical protein | - | 1287 | 428 | 570088…571374 |
| Mbr-GS01GM000492 | glycerol kinase | *glpK* | 1518 | 505 | 582597…584114 |
| Mbr-GS01GM000554 | lacI family transcription regulator | *scrR* | 972 | 323 | 653603…654574 |
| Mbr-GS01GM000565 | PTS system, glucose/glucosamine/beta-  glucoside-specific, IICBA component | - | 849 | 282 | 668405…669253 |
| Mbr-GS01GM000581 | ABC transporter, permease protein | - | 1233 | 410 | 696066…697298 |
| Mbr-GS01GM000584 | hypothetical protein | - | 360 | 119 | 701563…701922 |
