# Supplementary material for: Genome-Wide Analysis of Mycoplasma bovirhinis GS01 Reveals Potential Virulence Factors and Phylogenetic Relationships
Source: G3 (Bethesda). 2018 Mar 30;8(5):1417–24. doi: 10.1534/g3.118.200018 (PMC5940136; doi:10.1534/g3.118.200018)
Supplement: Supplementary file 1 [file 1417FileS1.zip › Supplementary Materials/Table S8 Genes in the genomic islands of M. bovirhinis GS01.doc]

**Table S8 Genes in the genomic islands of *M. bovirhinis* GS01**

| Locus | Product | Gene | Gene  length (bp) | Protein length (aa) | Position |
| --- | --- | --- | --- | --- | --- |
| genomics island from 463,870 to 481,877 | | | | | |
| Mbr-GS01GM000403 | RNA-binding protein S1 | - | 2151 | 716 | 461953…464103 |
| Mbr-GS01GM000404 | tRNA uridine 5-carboxymethylaminomethyl modification enzyme GidA | *mnmG* | 1830 | 609 | 464136…465965 |
| Mbr-GS01GM000405 | ribosomal RNA large subunit methyltransferase H | *rlmH* | 435 | 144 | 465965…466399 |
| Mbr-GS01GM000406 | type 11 methyltransferase | - | 723 | 240 | 466420…467142 |
| Mbr-GS01GM000407 | - | - | 1755 | 584 | 467132…468886 |
| Mbr-GS01GM000408 | DNA-directed RNA polymerase subunit beta | *rpoB* | 3624 | 1207 | 469093…472716 |
| Mbr-GS01GM000409 | DNA-directed RNA polymerase subunit beta' | *rpoC* | 4431 | 1476 | 472709…477139 |
| Mbr-GS01GM000410 | hypothetical protein | - | 3531 | 1176 | 477426…480956 |
| Mbr-GS01GM000411 | hypothetical protein | - | 1095 | 364 | 481551…482645 |
| genomics island from 561,137 to 565,821 | | | | | |
| Mbr-GS01GM000474 | amino acid permease | - | 1470 | 489 | 559085…560554 |
| Mbr-GS01GM000475 | serine-type ATP-dependent endopeptidase La (Lon) | *lon* | 2643 | 880 | 560556…563198 |
| Mbr-GS01GM000476 | NAD+ synthetase | *nadE* | 792 | 263 | 563499…564290 |
| Mbr-GS01GM000477 | transcriptional regulator | - | 735 | 244 | 564300…565034 |
| Mbr-GS01GM000478 | metal cation transporter, ZIP family protein | - | 1035 | 344 | 565034…566068 |
| genomics island from 597,644 to 607,572 | | | | | |
| Mbr-GS01GM000506 | - | - | 2511 | 836 | 595226…597736 |
| Mbr-GS01GM000507 | sucrose-specific PTS system IIBC component | *scrA* | 1569 | 522 | 597723…599291 |
| Mbr-GS01GM000508 | hypothetical protein | - | 372 | 123 | 600187…600558 |
| Mbr-GS01GM000509 | - | - | 225 | 74 | 600659…600883 |
| Mbr-GS01GM000510 | unknown protein | - | 297 | 98 | 604038…604334 |
| Mbr-GS01GM000511 | - | - | 417 | 138 | 604879…605295 |
| Mbr-GS01GM000512 | triosephosphate isomerase | *tpiA* | 735 | 244 | 605753…606487 |
| Mbr-GS01GM000513 | Holliday junction-specific endonuclease | *recU* | 483 | 160 | 606577…607059 |
| Mbr-GS01GM000514 | bacterial nucleoid DNA-binding protein | - | 291 | 96 | 607061…607351 |
| Mbr-GS01GM000515 | - | - | 1413 | 470 | 607468…608880 |
