# Supplementary material for: Genome-Wide Analysis of Mycoplasma bovirhinis GS01 Reveals Potential Virulence Factors and Phylogenetic Relationships
Source: G3 (Bethesda). 2018 Mar 30;8(5):1417–24. doi: 10.1534/g3.118.200018 (PMC5940136; doi:10.1534/g3.118.200018)
Supplement: Supplementary file 1 [file 1417FileS1.zip › Supplementary Materials/Table S9 The genome comparisons between M. bovirhinis GS01 and HAZ141_2 strains.doc]

**Table S9 The genome comparisons between *M. bovirhinis* GS01 and HAZ141_2 strains**

| Features | GS01 | HAZ141_2 |
| --- | --- | --- |
| Accession No. | CP024049 | AP018135.1 |
| Genome size (bp) | 847,985 | 948,039 |
| GC content | 27.57% | 28.24% |
| Protein-coding genes  (excluding pseudogenes) | 707 | 821^a^ |
| Protein-coding gene length (bp) | 757,599 | 840,594^a^ |
| Gene/Genome (%) | 89.34% | 88.67%^a^ |
| GC content in gene region | 27.96% | 28.67%^a^ |
| Gene average length (bp) | 1,072 | 1,024^a^ |
| Intergenic region length (bp) | 90,386 | 107,445^a^ |
| GC content in intergenic region | 24.30% | 24.88%^a^ |
| Intergenic length/Genome (%) | 10.66% | 11.33%^a^ |
| tRNA number | 31 | 31^a^ |
| rRNA (by de novo prediction) | 8 | 8^a^ |
| 5S rRNA (by de novo prediction) | 2 | 2^a^ |
| 16S rRNA (by de novo prediction) | 3 | 3^a^ |
| 23S rRNA (by de novo prediction) | 3 | 3^a^ |
| pseudogenes | 16^b^ | 38^b^ |

^a^ the data were predicted using the same method mentioned in GS01 genome;

^b^ the data were obtained from the NCBI GenBank database.
