# Supplementary figures and images for: Genome-Wide Analysis of Mycoplasma bovirhinis GS01 Reveals Potential Virulence Factors and Phylogenetic Relationships
Source: G3 (Bethesda). 2018 Mar 30;8(5):1417–24. doi: 10.1534/g3.118.200018 (PMC5940136; doi:10.1534/g3.118.200018)

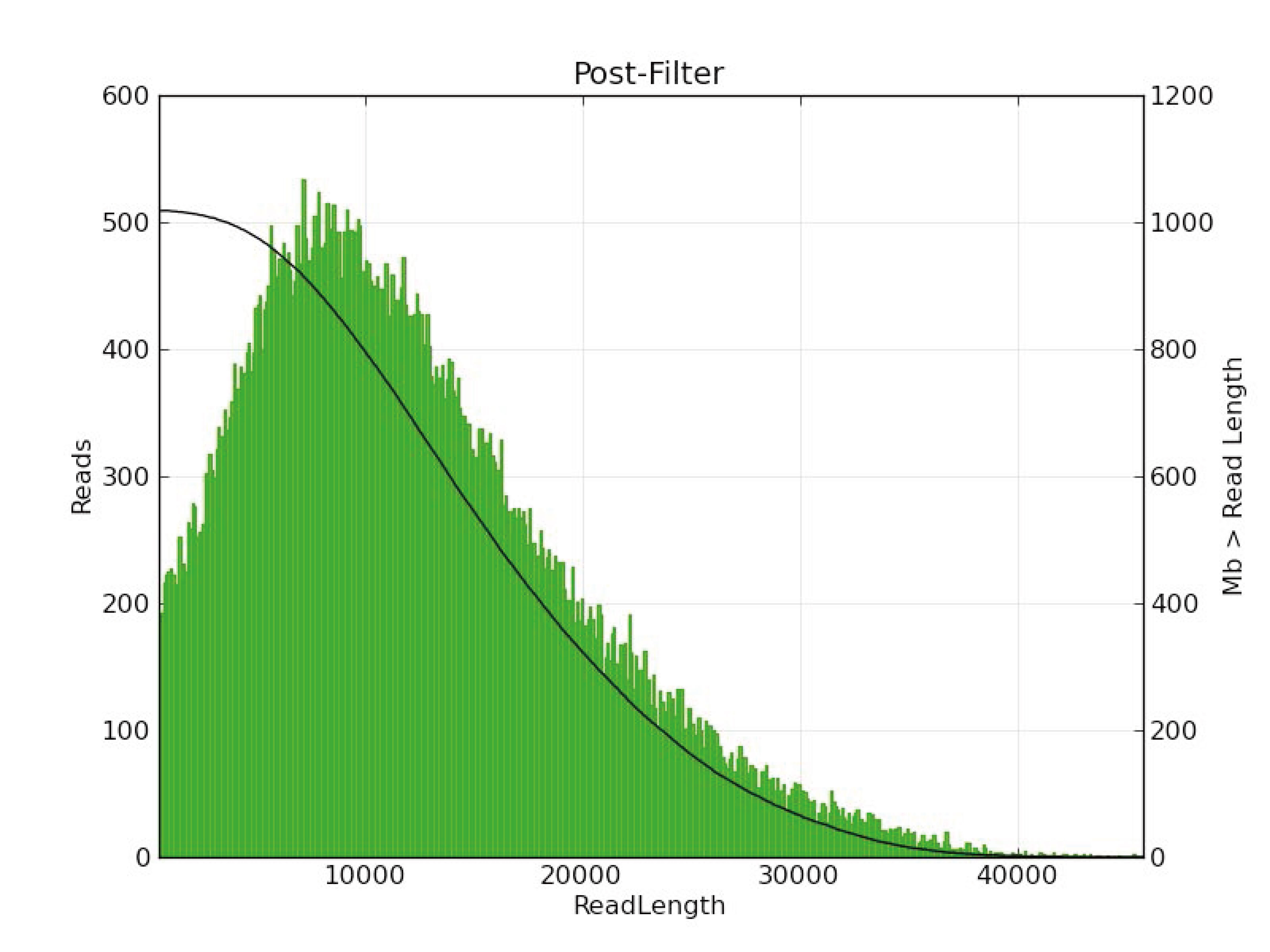

Supplement: Supplementary file 1 [file 1417FileS1.zip › Supplementary Materials/Figure S1 PacBio read length distribution for the sequenced M. bovirhinis GS01 genome.jpg]

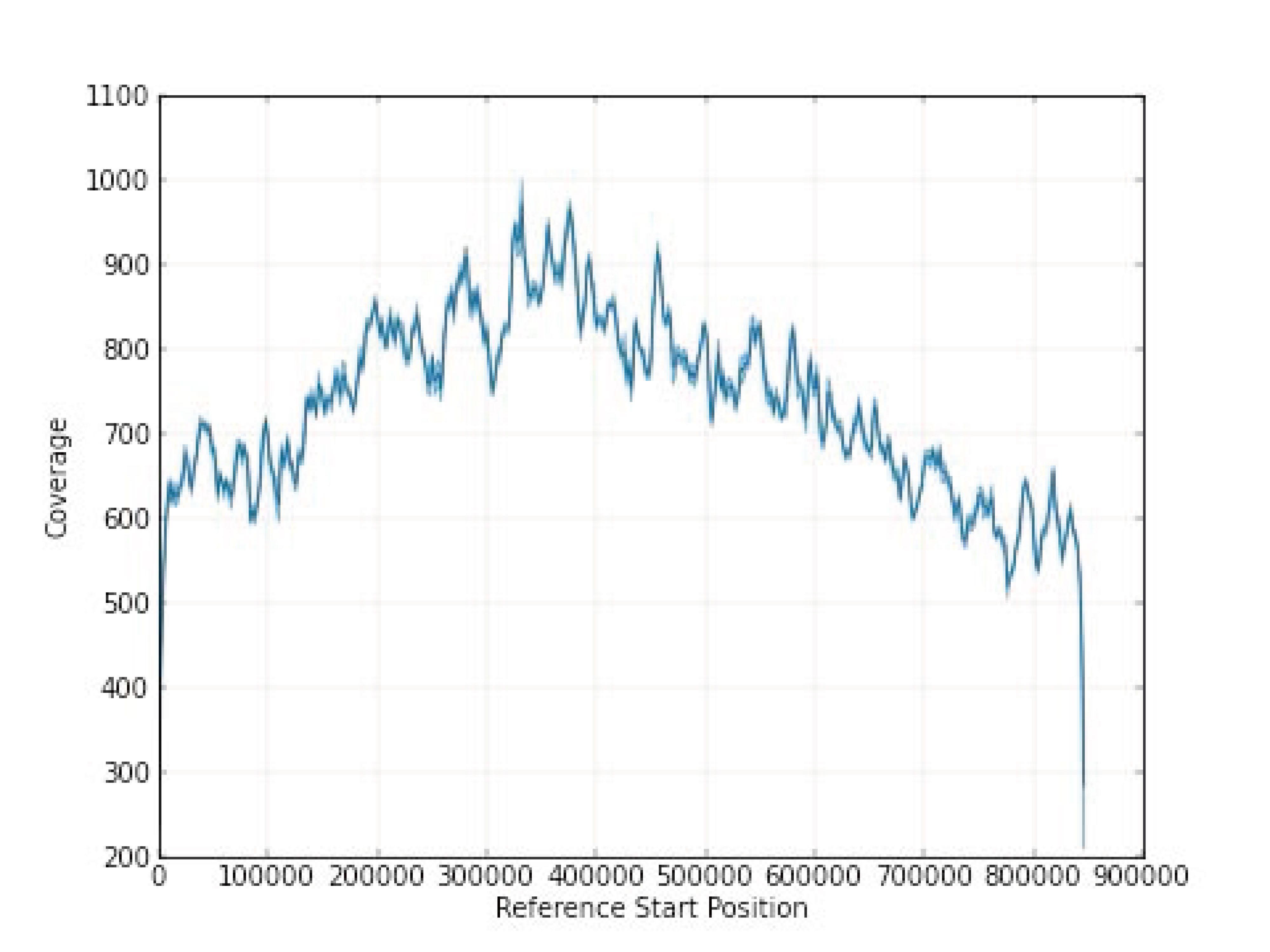

Supplement: Supplementary file 1 [file 1417FileS1.zip › Supplementary Materials/Figure S2 The depth of coverage distribution map for M. bovirhinis GS01 genome assembly.jpg]
